# Ecofriendly Extraction of Polyphenols from *Ampelopsis grossedentata* Leaves Coupled with Response Surface Methodology and Artificial Neural Network–Genetic Algorithm

**DOI:** 10.3390/molecules30112354

**Published:** 2025-05-28

**Authors:** Xubo Huang, Chen Li, Yanbin Wang, Jinrong Jiang, Weizhi Wu, Shifeng Wang, Ming Lin, Liang He

**Affiliations:** 1 Key Laboratory of State Forest Food Resources Utilization and Quality Control, Zhejiang Academy of Forestry, Hangzhou 310023, China; 13777834798@163.com (X.H.); 19884118285@163.com (C.L.); numbsword@126.com (Y.W.); weizhiwu197512@163.com (W.W.); linming1001@126.com (M.L.); 2 Forestry Technology Extension Station, Qingtian County Forestry Bureau, Lishui 323999, China; jiangjj1980@163.com (J.J.); shifengwang197010@163.com (S.W.)

**Keywords:** *Ampelopsis grossedentata* leaves, deep eutectic solvent, extraction, response surface methodology, artificial neural network

## Abstract

This study aimed to optimize a novel deep eutectic solvents (DESs)-assisted extraction process for polyphenols in the leaves of *Ampelopsis grossedentata* (AGPL) with response surface methodology (RSM) and a genetic algorithm–artificial neural network (GA-ANN). Under the influence of ultrasonic excitation, the L-carnitine-1,4-butanediol system was selected for the phenolics extraction process. The ideal conditions for AGPL extraction were the following: liquid to solid ratio of 35.5 mL/g, ultrasonic power of 697 W and extraction duration of 46 min. Under those conditions, the actual AGPL yield was 15.32% ± 0.12%. The statistical analysis showed that both models could predict AGPL yield well and GA-ANN had relatively higher accuracy in the prediction of AGPL output, supported by the coefficient of determination (R^2^ = 0.9809) in GA-based ANN compared to R^2^ = 0.9145 in RSM, as well as lower values for mean squared error (MSE = 0.0279), root mean squared error (RMSE = 0.1669) and absolute average deviation (AAD = 0.1336) in the GA-ANN model. Moreover, the extracted polyphenols were determined by HPLC-MS to have 20 phenolic compounds corresponding to some bioactive acids such as nonadecanoic acid and neochlorogenic acid. The in vitro ORAC assay revealed that Carn-Bu4 assisted AGPL extract exhibited a notable antioxidant capacity of 275.3 ± 0.64 μmol TE/g.

## 1. Introduction

Vine tea (commonly referred to as “Mei tea”, “Ratten tea”, or “Teng cha”) derived from the tender stems and leaves of *Ampelopsis grossedentata* Hand.-Mazz., VT, has been consumed as a popular folk tea in China for more than 600 years [1]. As first documented in the *Compendium of Materia Medica*, vine tea has attracted much attention due to its special mildly sweet flavor and cool properties compared to the prevailing traditional green tea, and is clinically used to treat many common diseases [2,3,4]. Moreover, a number of vine tea-related common foods and health foods have emerged after its legitimacy was acknowledged by the Chinese regulatory authority two years ago. Concerning the famous pharmacological features, those are mainly attributed to its rich bioactive components, including polyphenols, polysaccharides, proteins, and volatile components [5,6]. Despite being major components in the vine tea, some other polyphenols except dihydromyricetin [7] from *Ampelopsis grossedentata* Hand.-Mazz. (AGPL) have unfortunately been underestimated. Extensive studies indicate that polyphenols can take part in the normal regulation of surface membrane receptors and internal transcription factors to exert important pharmacological benefits including anti-oxidation, anti-inflammation, intestinal amelioration, immune regulation, etc. [8,9]. Nevertheless, little attentions has been focused on the extraction process of AGPL’s polyphenolic contents.

Some traditional techniques such as hot water extraction and ethanol assistance have been employed for such extraction, though with limited extraction rates and relative inefficiency [10]. Moreover, some types of phenolic compounds, including ethyl gallate, epicatechin and neochlorogenic acid, may not be influenced by such methods due to their weak combination with normal solvents. Ultrasonic waves can cause a cavitation effect by potent shear force and mechanical energy, which helps to release target compounds from the inner cell wall. Deep eutectic solvents (DESs), a promising chemical system, have been widely applied in extraction processes in many fields. DESs are a mixture of two or more substances that, when mixed, form a mixture with a lower melting point than any of the individual components, containing hydrogen bond receptors (HBA) and hydrogen bond donors (HBD) [11,12]. With unique biocompatibility, special tailorable solubility, excellent clearness and high efficiency, various procedures involving halogenated DESs have been reported for the extraction of some natural polyphenols [13]. Liu et al. [14] prepared choline-lactic acid as a good candidate for extracting polyphenols from *Cosmos sulphureus* with superior extraction performance. However, high halogen contents may increase the risk of corroding the extraction equipment to some extent [15]. Therefore, it is urgent to develop new halogen-free substances providing HBA for the DES system by improving the extraction efficiency. Yao et al. [16] selected a new halogen-free extractant to separate phenols from oils with high efficiency. Wang et al. [17] combined L-carnitine and formic acid to efficiently extract phenolics from coal tar.

Response surface methodology (RSM) and artificial neural networks have recently been utilized for the establishment of models for target yield prediction under various extraction conditions [18]. Both are statistical modelling tools used to analyze quantitative data by polynomial regression. RSM provides the optimal parameters via analysis of multivariate equations with greater precision and less processing time when compared to the one-factor-at-a-time method [19]. All the manipulations are within the range of second quadratic model predictions [20]. ANN has attracted great attention for its more suitable modeling. It is a cutting-edge multilayer perceptron network which searches globally for ideal conditions with the help of multiple non-linear functions [21]. Usually, proper training with input data with the assistance of the back-propagation algorithm would be a prerequisite for the establishment of an optimum network. With the combination of genetic algorithm (GA), the simulated network could produce satisfactory outputs by nonlinear mapping. This combination of GA coupled with ANN has been promoted in many extraction processes with satisfactory performance. Ramírez-Brewer et al. [22] reported that the maximum estimation of total phenolics content (121.3 mg GAE/g of extract) from mango peel was obtained using RSM and ANN predictions. Under the optimized output conditions predicted by ANN, the total polyphenol content from *Ecklonia radiata* could peak at 10.836 GAE mg/g.

The purpose of this study is to find a green and highly efficient solution to obtain polyphenol-rich extract from *Ampelopsis grossedentata* Hand.-Mazz. leaves. For that purpose, different DES constituents were employed to extract polyphenols from AGPL with ultrasonic assistance. A Box–Behnken experiment was then designed after the acquisition of single factor levels including water content, ultrasonic power, solid to liquid ratio, extraction temperature, etc. Based on the experimental data, a GA-ANN tool was used to optimize the simulated network by minimizing the squared errors. Moreover, the polyphenolic extract was subjected to high-performance liquid chromatography combined with mass spectroscopy (HPLC-MS) for its component determination. Finally, the antioxidant activities of the obtained AGPL extract under optimal conditions was evaluated by in vitro ORAC free radical assay.

## 2. Results

### 2.1. Screening of Suitable DESs System for AGPL Extraction

#### 2.1.1. The Optimal DESs System

**Figure 1:**
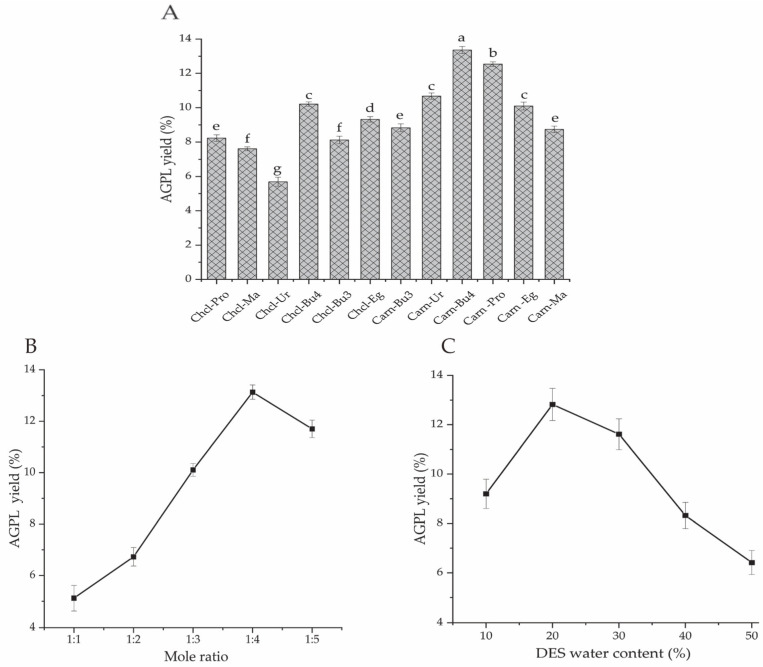
Comparative effects of different formulas on the extraction yield of AGPL (**A**); effect of molar ratio (**B**); effect of DES water content (**C**). (Bars with different letters differ significantly at *p* < 0.05).

Polyphenols derived from raw leaves of *A. grossedentata* were extracted by various DESs, which were designed to form a strong hydrogen bond in solution by different combinations of HBA and HBD [23]. As we all know, some DES formulations have little capacity to form uniform and transparent solvents, or cause precipitation of crystals in solution. This may be attributed to their poor hydrogen bonds. On the other hand, their special properties, including polarity, viscosity and solubility, can have remarkable impacts and affect the final extraction efficiency [24,25]. With regard to this point, we identified many successful combinations of HBA–HBD reported in the literature, and 12 DES solutions were prepared in this study to solubilize AGPL. Among them, choline chloride and L-carnitine were selected as two kinds of HBA, while 1,3-butanediol, urea, 1,4-butanediol, 1,2-propylene glycol, ethylene glycol, and malonic acid comprised HBD constituents. As shown in Figure 1A, the final AGPL yields fluctuated between 5.69 and 13.36% under the 12 DES conditions tested. It seemed that L-carnitine as HBA performed better in the AGPL extraction process than choline chloride, no matter what was used as HBD. That phenomenon might be explained by the fact that the Chcl-based solvents had relatively weak hydrogen bonds with the phenolic hydroxyl groups inside the polyphenol derivative, which were not appropriate for the phenolic compounds in vine tea. Moreover, the value of AGPL in Carn-Bu4 ranked top, with the highest production of 13.36% when compared with other formulas. This may be attributed to the moderate viscosity and polarity of Carn-Bu4 in the extraction process; under such circumstance, the target compounds experience reduced resistance at the inner cell wall due to hydrogen bonding forces [15]. Consequently, the Carn-Bu4 system could promote the extraction of AGPL from the leaves with higher binding affinity and was determined as the ideal DES formula for the subsequent experiments.

#### 2.1.2. Screening of Optimal Molar Ratio and Water Content

Certain chemicals might be precipitated if an inappropriate molar ratio of HBA to HBD occurs [26]. It is important to obtain the proper molar composition for the DES being used [27]. Figure 1B indicates that the highest AGPL yield (13.14%) was obtained when the molar ratio of L-carnitine to 1,4-butanediol was 1:4. Molar ratios larger or smaller than that had worse extraction rates for AGPL. This phenomenon indicates that the molar ratio could strongly affect the hydrogen bonding, Van Der Waals force and surface tension between all the phenolic fractions and extracts in DES solvent. The greater stability of Carn-Bu4 under such a molar ratio may promote the liberation of AGPL from the cell walls in the raw material.

Water content is another factor for DES which controls the polarity and viscosity of the solvent and accelerates the velocity of mass transfer [28]. It was noted that the highest AGPL yield was achieved when the water content was 20% (Figure 1C). After that, a remarkable decrease in production was found as the percentage of water content increased. This could be explained by the fact that large amounts of water in DES would attenuate, or even break, the hydrogen bond between HBD and HBA. In that case, setting an optimal water content for the Carn-Bu4 system with relatively lower viscosity should be beneficial for the liberation of the target phenolic substances. Moreover, similar polarity was found to be beneficial in solubilizing the phenolic derivatives [29]. Based on the above analysis, a water content of 20% and a molar ratio of 1:4 was finally determined as the suitable constituents of Carn-Bu4 during the AGPL extraction.

### 2.2. One-Factor-at-a-Time Evaluation

#### 2.2.1. Screening of Liquid to Solid Ratio

**Figure 2:**
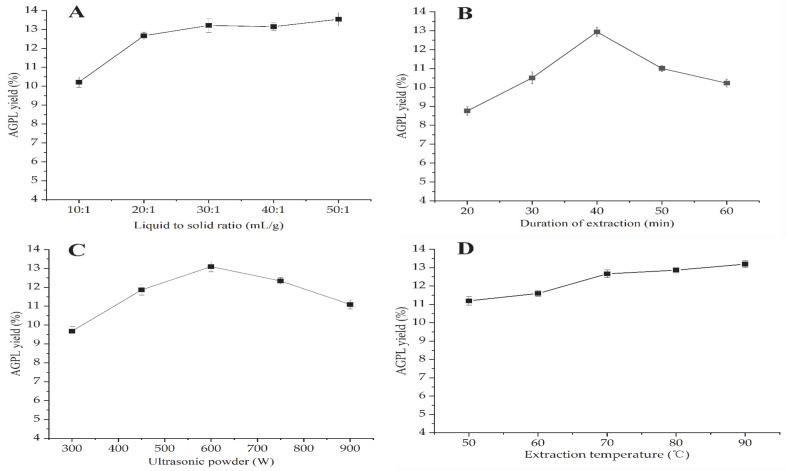
Single factors affecting AGPL yield by DES-based extraction. (**A**) Effect of liquid to solid ratio; (**B**) effect of duration of extraction; (**C**) effect of ultrasonic power; (**D**) effect of extraction temperature.

A series of liquid to solid ratios from 10:1 mL/g to 50:1 mL/g for extraction of AGPL were investigated (see Figure 2A). The results indicated that the maximum yield was achieved when the value of Carn-Bu4 to raw powder was 30:1 mL/g. Initially, the mass transfer and diffusion rate were sped up by full exposure of material particles to solvent, resulting in an increase of the extraction yield of up to 13.15%. After the addition of solvent into the mixture, that value was kept relatively stable due to insufficient solute; this is especially noticeable in the yield of 13.20% when the value was 50:1 mL/g. Too much solvent would be time-consuming for the next concentration step and increase the cost of the process. In order to reduce the follow-up cost, the range of 20:1–40:1 was selected for subsequent experiments.

#### 2.2.2. Screening of Extraction Duration

A prolonged extraction time can enhance the final yield to some extent. Figure 2B reveals that AGPL yield went up to a higher value—from 8.21% to 12.96%—with increasing time. It then dropped down slightly with further extension of the process, which reflects that in some stages, the Carn-Bu4 solvent exhibited steady performance in AGPL extraction. However, that balance was broken under ultrasonic treatment, inducing the decomposition of the extracted phenol compounds and the DES system [30,31]. It was necessary to calculate the optimal time under such a DES environment. Hence, the extraction times for the AGPL preparation were set to 30–50 min.

#### 2.2.3. Screening of Ultrasonic Power

Ultrasonic techniques have become an efficient and green tool to speed up the extraction process, and are popular in the fields of food production, pharmaceuticals and cosmetics [32]. Ultrasonic excitation can provide many beneficial functions such as powerful penetration of the solvent, motion frequency of the solute and high pressure. The cavity formed in the liquid by ultrasonic excitation may generate great pressure on the surface of the bubble, which drives the liberation of target compounds from the inner particles [33,34]. Figure 2C uncovers the dramatic increase of AGPL yield with from 300 W to 600 W ultrasonic treatment. Nevertheless, that assistance had the opposite effect on the extraction process. The reason for that may be ascribed to the decomposition of some unstable polyphenol substances and the covalent combination of Carn-Bu4. Too much ultrasonic energy may disturb the balance between HBA and HBD or polyphenols. So, it was necessary to investigate the ideal value of ultrasonic power in the next RSM optimization.

#### 2.2.4. Screening of Extraction Temperature

As an agitating force, temperature is generally recognized as the key factor to strengthen the driving force of DES solvent on the solubility of the target molecules [35]. This study tested the impact of five extraction temperatures on AGPL contents, and the results are shown in Figure 2D. Contrary to expectations, AGPL production showed a steady plateau during the whole process, which did not increase significantly compared to the first value of 11.05%. A similar phenomenon has been found in other reports [36]. This finding further evidenced the function of ultrasonic waves in the process of extraction, as they may provide enough energy for the extraction process and correspondingly attenuate the function of temperature. Concerning the application of ultrasonic waves as the new promotor, it was undesirable to provide too high a temperature. With respect to the double action of ultrasonic waves on the system, i.e., reducing the viscosity of DES and saving energy, the extraction temperature was kept at 60 °C for the whole process.

### 2.3. Optimization by RSM

#### 2.3.1. BBD Analysis

BBD based on RSM was employed to analyze the influence of independent variables (Liquid to solid ratio (X_1_), Ultrasonic power (X_2_) and Duration of Extraction (X_3_)) on the dependent target (AGPL yield). Table 1 describes 17 combinations of experimental inputs based on a multilevel-factorial design, and each output value was tested in triplicate and in parallel. using DesignExpert 12, a reliable polynomial equation was simulated to predict the final yield and correlate those data accurately, which is presented as follows:

y = 14.88 + 0.6100X_1_ + 0.0600X_2_ + 0.1325X_3_ − 0.0625X_1_X_2_ − 0.0625X_1_X_3_ − 0.4925X_2_X_3_ − 0.3313X_1_^2^ − 0.5013X_2_^2^ − 6262X_3_^2^ (1)

The statistical significance of Equation (1) was checked using Fisher’s statistical ANOVA test (F-test) and results are shown in Table 2 with the labels * and ** in the last column. The results indicated that the second model was well-suited for the prediction of experimental data, supported by a highly significant model F value of 8.92 and a low *p* value of *p* = 0.0043. The value of R^2^ (0.9145) shows close agreement between the experimental results and the theoretical values predicted by the polynomial model. The adjusted coefficient of determination (R^2^_adj_) indicated that the sample variation of 81.67% for the AGPL yield was attributable to the independent variables. Moreover, a relatively low value of CV (2.18) further illustrated that the experiments were practical with better precision and reliability [37]. The non-significant lack of fit (*p* = 0.082) indicated the good fit of this model for prediction. The coefficient estimates of Equation (2), along with the corresponding *p*-values, are presented in Table 1. It can be seen that the *p* values of X_1_, X_2_, X_1_X_2_, X_1_X_3,_ and X_3_^2^ were all less than 0.05, showing extremely significant levels except for X_3_ and X_2_X_3_ (*p* > 0.05). The effects of three parameters on the AGPL yield followed this order: ultrasonic power > liquid to solid ratio > duration of extraction.

#### 2.3.2. Interactive Effects on AGPL Yield

**Figure 3:**
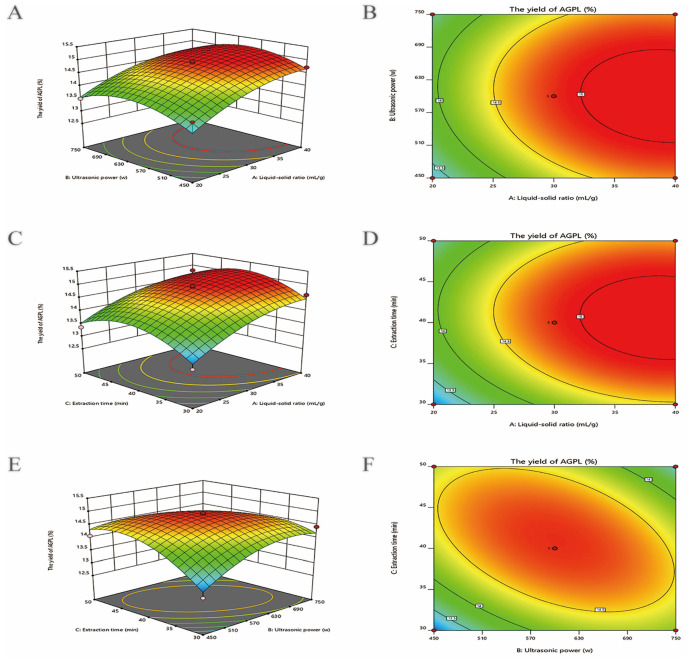
The effects of the interactions among three experimental factors on AGPL yield: three-dimensional surface plots (**A**,**C**,**E**) and two-dimensional contour maps (**B**,**D**,**F**).

The fitted response surface plots and their corresponding contour plots for DES-based extraction of AGPL by the BBD model are shown in Figure 1, which provides a visualization of the relationship and interaction between the response and experimental levels of each variable. Figure 3A indicates that the interaction between X_1_ and X_2_ had a pronounced impact on the AGPL yield at a significant level (*p* = 0.0082). It was evident that when the liquid to solid ratio was from 20 to 40 mL/g, and the ultrasonic power was from 450 to 630 W, the AGPL yield was over 13.67% and then decreased slowly beyond this range. The reason was ascribed to the enhancement of ultrasonic power at the initial stage, promoting the process of extraction. With exposure to higher levels of ultrasonic waves, the prepared DES system would break and result in lower efficiency of the process. Moreover, the increase of the liquid to solid ratio to some extent could improve the concentration difference and promote the solubility of the target. Those facts were consistent with the results for *Ilex latifolia* polyphenols [38], in which the factors of ultrasonication and liquid to solid ratio were shown to break down the plant cell wall and draw the flavonoids out of the inner cell.

The interaction of increasing liquid to solid ratio and duration of extraction had an obvious influence (*p* = 0.0153) on the AGPL yield in Figure 3B when X_2_ was set to zero. At the liquid to solid ratio of 30 mL/g, the duration of extraction was beneficial to the enhancement of AGPL yield. The highest yield of 14.6% was obtained when the extraction time was less than 45 min. After that, the final outcome had a negative response to increasing liquid to solid ratio and extraction time. This result was consistent with the findings of Jiao et al. [39], who reported that an optimal duration for the process would help choline chloride–acetic acid to extract flavonoids form *Perilla frutescens* leaves, whereas a prolonged extraction time led to degradation of flavonoids by hydrolysis of the O-glycosidic bonds.

Figure 3C shows that the yield of AGPL climbed continuously when the value of X_2_ increased from 450 W to 600 W and that of X_3_ extended from 30 min to 42 min. The yield dropped dramatically outside of that optimum point. The contour plot was diagonally elliptical, revealing that the mutual influence of ultrasonic power and duration of extraction had a relatively weak effect (*p* < 0.6978). With the assistance of ultrasonic waves for the initial process of AGPL extraction from leaves of *A. grossedentata*, the diffusion process was enhanced and components were forced into the solvent more rapidly during the extraction process. However, it had no effect on the yield of AGPL at higher ultrasonic power level and duration, which might be explained by the solubility of impurities and a decrease in the available surface area between Carn-Bu4 solvent and the cells. Those findings can also be found in the report of Zhang et al. [23].

### 2.4. ANN Model Establishment

**Figure 4:**
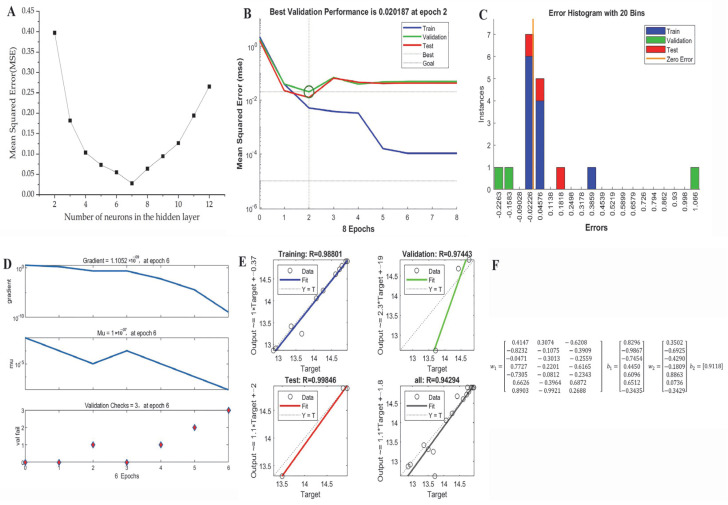
The relationship between MSE and the number of hidden neurons (**A**); the performance of the ANN model for training, validation and test (**B**); error histogram with 20 bins (**C**); the training state (**D**), regression analysis (**E**) and the weights and bias matrices of the topology (**F**).

A three-layered neural network was constructed to simulate the extraction process of AGPL under various experimental conditions. To achieve that, the structure of the multilayer perceptron framework was first established, which was determined by the number of neurons in the hidden layer. As depicted in Figure 4A, as the number of hidden neurons changed, the MSE value showed a concave curve during the training of various ANN topologies. The minimum value of MSE was 0.0256 when the number of neurons was 7. Hence, the ANN topology of 3-7-1 was the ideal structure for that prediction, where a 70% subset was used for training, 15% of the variables were used for validation and the remaining 15% was used for testing. Prior to that manipulation, the 3 × 7 weight matrix of the input layer connected to the hidden layer and 7 × 1 weight matrix of the hidden layer to output layer were assigned in the following w_1_ and w_2_, where the related bias matrixes of b_1_ and b_2_ were also present for the model calculation [40].

Figure 4B shows that the best evaluation performance was 0.020187 MSE value at 2 epochs. A significant drop of MSE occurred in the training step, but the value remained stable in the validation and test steps, which suggested overfitting. Figure 4C presents a reasonable range for data fitting errors, with the highest instance near zero. The values of gradient, Mu and val fail were 1.1052 × 10^−9^, 1.0 × 10^−7^ and 3 at 6 epochs, respectively, suggesting that the ANN model is well-trained (Figure 4D). The good correlation between predicted and actual values is demonstrated by the four linear relationships in Figure 4E, where the R values of training, validation, test and all were 0.99947, 0.95222, 0.99998 and 0.988, respectively. All the weights and bias matrices of the 3-7-1 topology were determined by the calculation in Figure 4F.

### 2.5. GA-Oriented Optimization

**Figure 5:**
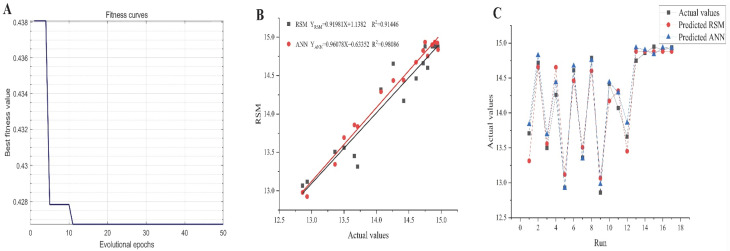
The optimization process by GA-oriented ANN (**A**); the relationship between the predicted and actual values provided by RSM and ANN (**B**); the matching plot between two methods and all datasets (**C**).

Renowned for its global search capability and adaptive control, the GA is widely employed in solving complex optimization problems [41]. In this study, the hybrid ANN-GA approach was utilized to determine the optimal extraction parameters for AGPL. The individual chromosome contained three input variables and the fitness function was employed to test the fitness of every solution when the chromosome population was enlarged to 1000. The selected chromosomes with high performance were sent to replicate new generations through crossover (0.8) and mutations (0.2). That evolutional process was iterated until the optimal output emerged. As depicted in Figure 5A, the GA performed 100 iterations to identify the most efficient extraction conditions, ultimately yielding the highest AGPL content. During the first 20 generations, a substantial decrease in MSE was observed, demonstrating the algorithm’s ability to rapidly locate a superior candidate solution within the search space—a testament to its strong initial optimization performance. From the 20th to the 100th generation, the GA reached convergence, indicating that an optimal or near-optimal solution had been identified, with further iterations providing no significant enhancement. The ANN-GA model predicted the following optimal conditions: liquid to solid ratio of 35.4922 mL/g, ultrasonic power of 696.7965 W and extraction duration of 46.1238 min. For practical implementation, these parameters were adjusted to 35.5 mL/g, 697 W and 46 min, respectively. Experimental validation under these modified conditions yielded an AGPL content of 15.32% ± 0.12%, closely aligning with the model’s prediction (15.2822%). These findings confirmed that the ANN-GA model exhibited strong predictive accuracy and could reliably estimate AGPL yield based on specified extraction parameters.

### 2.6. Comparative Analysis of RSM and ANN

The plot tendency of predicted values and experimental data provided by the RSM and ANN mathematical models indicated that both of the models have a good degree of fitness (the red line and black line) in Figure 5B. However, the higher value (0.9809) of coefficient of determination (R^2^) in the GA-based ANN indicated that its prediction ability was more accurate than that of RSM (R^2^ = 0.9145) in Table 3, which meant the former had 98.09% confidence in explaining the changes in the corresponding AGPL yield. It can be seen that all the predicted ANN points more closely approached the actual data (Figure 5C). Moreover, all values were lower than those in the RSM model (0.0393, 0.1982 and 0.1641) in Table 3, demonstrating that the GA-based ANN model could accurately simulate the relationship between AGPL yield and three selected variables. This prominent accuracy was ascribed to the excellent non-linear processing, fault tolerance, self-learning, self-training and global searching provided by the GA-ANN mathematical tool [42]. With that prediction, the halogen-free L-carnitine-But4 system could display a pronounced contribution in terms of polyphenols extraction, comparable to the phenolic yield offered by choline chloride based DESs [12,13].

### 2.7. The Qualitative Analysis of UPLC-ESI-QTOF-MS

**Figure 6:**
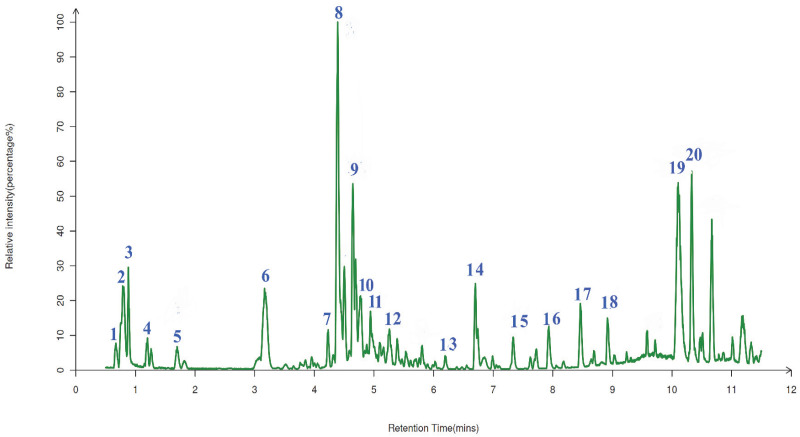
Total ionic profile of AGPL detected by UPLC-ESI-QTOF-MS/MS determination.

The phenolic compositions of AGPL were qualitatively analyzed by UPLC-ESI-QTOF-MS. The molecular formula and fragmentation patterns were provided by MS/MS double detection with a comparison of MS databases including the Human Metabolome Database (https://www.hmdb.ca/, accessed on 12 March 2024), LipidMaps (https://lipidmaps.org, accessed on 12 March 2024), mzcloud (https://www.mzcloud.org, accessed on 12 March 2024). There were 20 phenolic compounds found in the leaves of *Ampelopsis grossedentata*, shown in Figure 6 and listed in Table 4. Among them, eight major phenolic items were identified with the help of positive and negative ionization modes. Those were Leucocyanidin (*m*/*z* = 306), Quercetin (*m*/*z* = 303), (−)-Epigallocatechin (*m*/*z* = 307), Quercetin 3-O-rhamnoside 7-O-glucoside (*m*/*z* = 609), Nonadecanoic acid (*m*/*z* = 297), Quercetin 3-O-glucoside (*m*/*z* = 463), Dihydromyricetin (*m*/*z* = 319) and myricitrin (*m*/*z* = 463). Dihydromyricetin was found to have a significant peak at RT = 10.07 min, which agreed with the finding that it is the most important compound in vine tea. In addition, some bioactive acids were successfully liberated from the inner part of the raw leaves due to the contribution of the novel Carn-Bu4 system in the extraction process. This can be attributed to the combination of two HBD-HBA sources, which may strengthen the hydrogen bonding force in the solvent.

### 2.8. Antioxidant Activity Analysis of AGPL In Vitro

**Figure 7:**
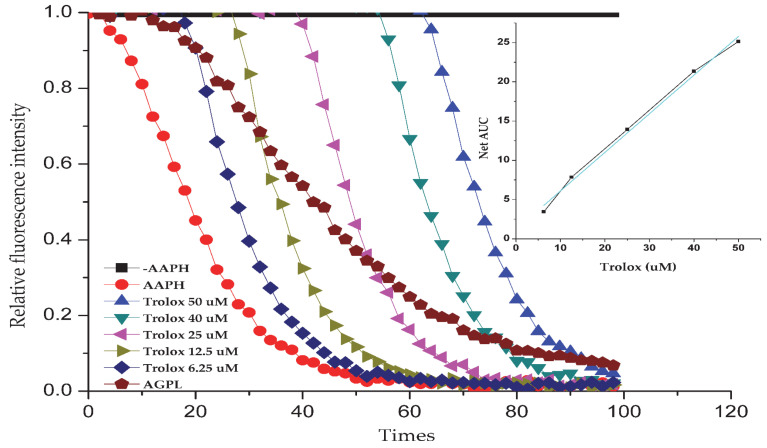
Fluorescence consumption induced by AAPH-derived radicals in the presence of Trolox ranging from 6.25 to 50 μM and AGPL with pH 7.4 phosphate buffer. Note: the profile of NetAUC versus Trolox was presented in the right corner.

Oxygen radical absorbance capacity (ORAC) has been proved to effectively and easily quantify the antioxidant capacity of drugs. It is conducted based on the chemical damage to β-PE caused by a peroxyl radical-producing compound (AAPH), reducing the fluorescence emission of β-PE. Due to the existence of antioxidants in the medium, the damage can be repaired to some extent, and thus the reduction in the fluorescence emission is prolonged. In this study, a calibration curve was constructed with Trolox concentration (μmol/L) as the abscissa and NetAUC (net area under the curve) as the ordinate [43]. As shown in Figure 7, the linear regression equation was determined as y = 0.8981x + 2.8097 (R^2^ = 0.9572), demonstrating satisfactory linearity. The NetAUC values of AGPL were interpolated into this standard curve to calculate corresponding Trolox-equivalent concentrations. The oxygen radical absorbance capacity (ORAC) was expressed as Trolox equivalents (μmol/g dry weight). Based on the calibration curve, the Carn-Bu4 assisted AGPL extract exhibited an ORAC value of 275.3 ± 0.64 μmol TE/g (mean ± SD, *n* = 6), indicating substantial antioxidant activity. These data suggest that AGPL extract could be an effective electron donor capable of reacting with free radicals to convert them into more stable products.

## 3. Materials and Methods

### 3.1. Materials and Reagents

The fresh leaves of *Ampelopsis grossedentata* Hand.-Mazz. were collected from Dayang Mountain, Qingtian County, Zhejiang Province in April. All reactants and solvents were of analytical grade. The gallic acid standard (purity >98%) was purchased from Sigma-Aldrich (Shanghai, China). Choline chloride, L-carnitine, 1,4-butanediol, 1,3-butanediol, ethylene glycol and ethanol were provided from Sinopharm Chemical Reagent Co., Ltd. (Shanghai, China). Distilled water was produced using a Kebang water treatment system (Kebang 210B, Hangzhou, China). Folin–Ciocalteu reagent, TPTZ [1,3,5-tri (2-pyridyl)-2,4,6-triazine], 2,2′-azino-bis-(3-ethylbenzothiazoline-6-sulfonic acid) diammonium salt (ABTS), 2,2′-azobis (2-methylpropionamidine)-dihydrochloride (AAPH), fluorescein sodium salt, 6-hydroxy-2,5,7,8-tetramethylchroman-2-carboxylic acid (Trolox) were purchased from Sigma-Aldrich Chemical Co. (St. Louis, MO, USA). All other chemicals were at analytical grade.

### 3.2. Preparation of DESs Formula

Two kinds of formula were adopted to constitute the hydrogen bond acceptors and hydrogen bond donors of the DESs. Choline chloride and L-carnitine were selected for the HBAs, while different alcohols, malonic acid and urea were chosen as the HBDs. All the uniform solutions were strictly prepared by constant stirring and heating in an oil bath at 80 °C, followed the recipes listed in Table 5. After cooling, the transparent liquids were ready to be used in the following extraction process.

### 3.3. DESs-Based Extraction and Contents Determination of AGPL

Prior to the extraction, leaves (freeze-dried for at least 36 h) of *A. grossedentata* were smashed into small particles through a 100-mesh sieve. Then 2.000 g of powder in a glass conical flask was mixed with various concentrations of DESs solvents or water and treated with ultrasonication at various power levels (300–900 w) in a water bath at 40–60 °C for the extraction process. After centrifugation at 8000× *g*, the supernatant was transferred to a 100 mL volumetric flask where the volume was made up to 100 mL. Then, the polyphenols in the DES were recovered by the anti-solvent method. Distilled water was added to the extract solution at a ratio of 20:1 (*v*/*v*) and the mixture was held at 4 °C for 1 h. Centrifugation at 6000 rpm for 10 min followed to obtain the final precipitate, which was collected and dried to a constant weight. The water-rich DES was evaporated by vacuum reduction [44].

For the content measurements of phenolic compounds by the Folin–Ciocalteu colorimetric method, a standard stock solution of gallic acid (0.234 mg/mL) was prepared, and aliquots of 0.0, 0.1, 0.25, 0.5, 0.75, and 1.0 mL were accurately pipetted and diluted to 2 mL with deionized water. Subsequently, 0.5 mL of Folin–Ciocalteu reagent was added to each mixture, followed by thorough vortexing and incubation at room temperature for 3 min. Then, 4 mL of 7.5% (*w*/*v*) sodium carbonate (Na_2_CO_3_) solution was added, and the reaction mixture was vigorously mixed and allowed to stand for 60 min at ambient temperature. After the auto zero adjustment, a UV-Vis spectrophotometer (Hitachi 1000, Tokyo, Japan) was used to record the absorbance values (A) of all samples at 760 nm. Experimental data were analyzed to establish a linear regression equation (y = 16.784x − 0.0218, R^2^ = 0.9992), where the y-axis represents absorbance and the x-axis corresponds to gallic acid concentration. The percentage yield of polyphenols in leaves of *A. grossedentata* (Y, %) was calculated by the following equation:

Y = C × V × r/W(2)

C means the tested polyphenol concentration in the tube (g/mL), V represents the volume of extraction (mL), r is the dilution ratio and W refers to the dry weight of raw material (g).

### 3.4. The Effects of Molar Ratio and Water Content on Polarity of DESs

In order to improve the polar properties of the DES system for maximum polar/nonpolar polyphenolics extraction, different molar ratios (1:1, 1:2, 1:3, 1:4 and 1:5) of the selected DES solvent with a series of water contents (10%, 20%, 30%, 40%, and 50%) were tested for the acquisition of the highest AGPL yields when the other conditions were constant.

### 3.5. RSM-Based Model of AGPL Extraction Process

For the optimization of the target extraction, four different single factors were immediately investigated before the RSM modeling, which were liquid–solid ratio (10:1 mL/g, 20:1 mL/g, 30:1 mL/g, 40:1 mL/g and 50:1 mL/g), duration of extraction (20 min, 30 min, 40 min, 50 min and 60 min), extraction temperature (50 °C, 60 °C, 70 °C, 80 °C and 90 °C), and ultrasonic power (300 W, 450 W, 600 W, 750 W and 900 W). Each experimental condition was tested three times to obtain an average value and presented as mean ± standard deviation (SD).

A Box–Behnken design (BBD) (Design Expert Software, trial version 7.1.3; Stat-Ease Inc., Minneapolis, MN, USA) was used to determine the best combination of extraction variables for production of AGPL from the leaves of *A. grossedentata*. Three variables were established on the basis of “one-factor-at-a-time” trials for AGPL yield (Table 6). The design included 17 experimental trials (Table 2). A total of five replicates at the center of the design were used to allow for estimation of a pure error sum of squares. Each experiment was performed in triplicate and the yield of AGPL (%) was interpreted as the response (Y).

### 3.6. ANN Model

**Figure 8:**
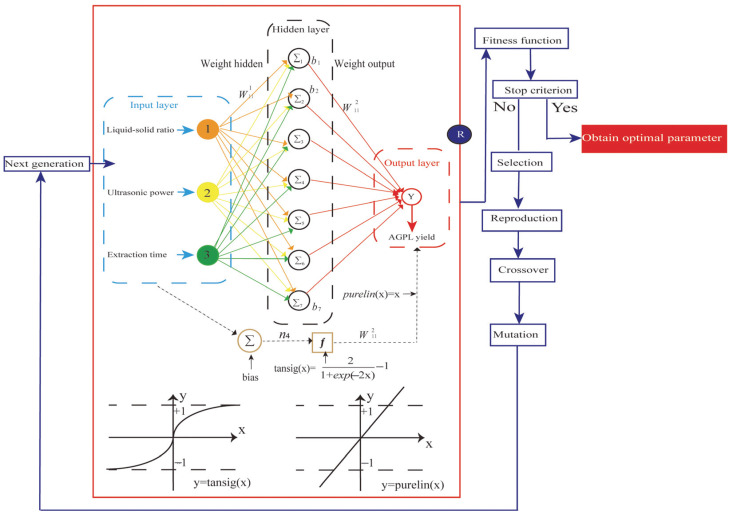
The topology of ANN architecture (input layer, hidden layer and output layers) in red, followed by the GA-based optimization (blue parts).

The Matlab 2020a software package was used to construct an ANN model based on the experimental results of AGPL yield designed by RSM. As depicted in the left frame of Figure 8, the architecture of the artificial neural network model was initially established by training the independent variables and testing the dependent variables [45]. For this study, the model consisted of an input layer (X_1_, X_2_, X_3_), a hidden layer, and an output layer (MPTGL yield). Prior to the simulation, all the data sets were categorized into three parts: training (70%), validation (15%), and testing (15%). Then, the neural network model was trained iteratively until the mean of error between the experimental and predicted values of AGPL yield reduced to its minimum. The hyperbolic tangent sigmoid function (tansig) was used for the communication between input layer and hidden layer while the linear function (purelin) was selected for the combination of hidden layer and final outcomes. Within the ANN model, the hidden nodes were the crucial parameters which significantly affected its topology and predictive capacity. In this study, 10 neurons were found to be suitable for the neural network prediction under the back-propagation Levenberg–Marquardt algorithm by controlling the weights and biases. Due to the discrepancy with actual values, all experimental results were standardized between −1 and 1 by Equation (3). These normalized values were subsequently converted back to actual values after processing through the output layer of the network [46].

(3) $$M_{i}=\frac{\left(M_{max}-M_{min})(N_{i}-N_{min}\right)}{N_{max}-N_{min}}+N_{min}$$

where M_i_ is the normalized value, M_max_ and M_min_ are the maximum and minimum values of the scaling range, and N_i_ is the actual data to be normalized. N_max_ and N_min_ are the maximum and minimum values of the actual data.

Other training parameters were as follows: training epochs, learning rate, goal, gradient and failure were 1000, 0.1, 0.00001, 1 × 10^−6^ and 6, respectively. After the modeling, the well-trained ANN was transformed into a mathematical equation by combining the following transfer function:

(4) $$Y(\%)\;=\;purelin\;\left(\left[\sum_{i=1}^{N}w_{i}^{2}tansig\left(\sum_{j=1}^{j}w_{ij}^{1}X_{j}+b_{1i}\right)\right]+b_{2}\right)$$

(5) $$tansig(x)=\frac{2}{1+exp\left(-2x\right)}-1$$

purelin (x) = x (6)

where x and j are the experimental factors (the input variables) and the number of input variables, respectively. w_1_ and b_1_ are the weight and bias of the hidden layer, respectively. w_2_ and b_2_ are the weight and bias of the output layer, respectively.

### 3.7. Model Analysis

Four classical indicators were employed to evaluate the prediction performance of RSM and ANN, which were values of R^2^, mean squared error (MSE), root mean squared error (RMSE), and absolute average deviation (AAD) with the equations as follows:

(7) $$R^{2}=1-\frac{\sum_{i=1}^{n}{\left(X_{i}-X_{ik}\right)}^{2}}{\sum_{i=1}^{n}{\left(X_{ik}-X_{z}\right)}^{2}}$$

(8) $$MSE\;=\frac{1}{n}\sum_{i=1}^{n}{\left(X_{i}-X_{ik}\right)}^{2}$$

(9) $$RMSE\;=\sqrt{\frac{1}{n}\sum_{i=1}^{n}{\left(X_{i}-X_{ik}\right)}^{2}}$$

(10) $$\;AAD\;\left(\%\right)=\left[\frac{\sum_{i=1}^{n}\left(\left|X_{ik}-X_{i}\right|/X_{ik}\right)}{n}\right]\times 100$$

where X_i_ is the predicted APGL yield, X_ik_ is the experimental or actual APGL yield, X_z_ is the mean of experimental APGL yield, and n represents the number of parameters in each model.

### 3.8. Optimization of the Extraction Process

In order to avoid the dilemma of local optima, the genetic algorithm method was introduced to help the neural network search for the optimal extraction parameters of AGPL [47]. Like the learning principle of biological evolution, a series of manipulations of species reproduction, crossover, mutation and selection were conducted to vary the fitness values in the trained BP network. Then, the maximum yield was achieved by converting a function to an inverse function or by changing the sign. Based on MATLAB 2020a, a GA toolbox was adopted to simulate those natural evolution processes. The population size, crossover fraction, mutation ratio, and generations were set as 100, 0.8, 0.1 and 300, respectively. Other parameters were given their default values. The above ANN-derived Equation (3) was introduced as a fitness function. The higher the AGPL production, the greater the function value of the individual.

### 3.9. Phenolic Composition Determination by UPLC/ESI-QTOF-MS/MS

According to the method reported by Wang et al. [48], AGPL solid extract was dissolved into 600 μL of methanol containing 2-chloro-L-phenylalanine (4 ppm), followed by vortex mixing for 30 s. The mixture was then sonicated at room temperature for 15 min and centrifuged at 12,000 rpm (4 °C) for 10 min to get the supernatant. After filtration through 0.22 μm membrane, the sample was injected into an autosampler vial for measurement by an ACQUITY UPLC HSS T_3_ (50 mm × 3.0 mm, 1.8 μm, Thermo Fisher Technologies Co., Waltham, MA, USA) coupled with Triple-TOF 5600^+^ Mass Spectrometry (AB SCIEX Co., Framingham, MA, USA). The conditions were the following: The column temperature was maintained at 40 °C. The mobile phase consisted of acetonitrile (A) and 10 mM ammonium formate aqueous solution (0.1%, B) with a flow rate of 0.3 mL/min. The injection volume was set to 2 μL. The elution procedure was performed as follows: 0–1 min, 0% to 8% B; 1–8 min, 8–98% B, 8–10 min, 98% B; 10–10.1 min, 98–8% B, 10.1–12 min, 8% B. The MS conditions were set as positive/negative ion sweeping mode: capillary voltage 4 KV, capillary temperature 325 °C, scan range 100–1000 *m*/*z* with sweep resolution of 60,000, ion source gas 1 and 2 (air) 55 psi, curtain gas (N_2_) 35 psi, ion source temperature 600 °C (positive) and 550 °C (negative), source voltage 3.5 kv (positive) and −2.5 kv (negative), collision energy was set to 20 eV. Data were processed using Analyst software version 4.1 (MassLynx).

### 3.10. In-Vitro ORAC Activities of AGPL

We prepared 96-well fluorescent plates to examine the experimental samples according to the method described in [49]. In order to eliminate the possibility of cytotoxicity of residual DES in the extract, the final samples were obtained by anti-water precipitation in DES as described in Section 3.3. All groups were simultaneously supplemented with 40 μL of fluorescein salt (700 nM). Subsequently, 20 μL of phosphate buffered saline (PBS) was added to the control group, 20 μL of water-soluble VE derivative Trolox (200 μM) to the standard curve group, and 20 μL of test sample to the sample group. The plate was incubated at 37 °C for 15 min. Following incubation, 140 μL of PBS was added to the negative control group, while 140 μL of AAPH (12 mM) was added to all other test wells. Fluorescence intensity was immediately measured using a microplate reader under 485 nm excitation wavelength, with emission recorded at 538 nm. Measurements were conducted at 2 min intervals from 0 to 120 min. The area under the curve (AUC) of the zero-order moment for fluorescence intensity changes was calculated using Equation (1). A Trolox standard curve was plotted to determine the oxygen radical absorbance capacity (ORAC) value of the samples.

(11) $$AUC=1+f_{1}/f_{0}+f_{2}/f_{0}+f_{3}/f_{0}+ï+f_{n}/f_{0}$$

(12) $$ORAC=\frac{{AUC}_{sample}-{AUC}_{+AAPH}}{{AUC}_{Trolox}-{AUC}_{+AAPH}}\times \frac{n_{Trolox}}{n_{Trolox}}$$

where f_0_ is the initial fluorescence value at 0 min and f_i_ the fluorescence read at i min.

### 3.11. Statistical Analysis

The data were expressed as mean ± standard error. Statistical differences were calculated using IBM SPSS 19.0 based on Turkey test with a confidence level of 95% as significant and a confidence level of 99% as extremely significant.

## 4. Conclusions

In this study, an ecofriendly and effective DES system consisting of L-carnitine-1,4-butanediol in the molar ratio of 1:4 with 20% water content was conducted to extract polyphenols from the leaves of *Ampelopsis grossedentata*. In order to avoid overfitting when searching for the optimal solution, a genetic algorithm–artificial neural network was employed to make the prediction based on reasonable experimental data designed by RSM. After training, validation and testing, the optimal conditions were established as follows: liquid to solid ratio of 35.5 mL/g, ultrasonic power of 697 W and extraction duration of 46 min. With those parameters, the ideal AGPL yield was 15.32% ± 0.12% with a higher R^2^ = 0.9809 in the GA-based ANN model compared to R^2^ = 0.9145 in RSM. Considering the lower MSE (0.0279), RMSE (0.1669) and AAD (0.1336), GA-ANN model exhibited better performance in prediction than RSM. Subsequently, bioactive acids including nonadecanoic acid and neochlorogenic acid were extracted along with 18 other phenolic substances by the carn-but4 system, which were determined by HPLC-Q-TOF-MS/MS. The extract had potent antioxidant activity, with an ORAC value of 275.3 ± 0.64 μmol TE/g. These findings could accelerate the extraction of polyphenols from the leaves of *Ampelopsis grossedentata* and highlight the advantages of nonlinear multilayer perception networks for process optimization.

## Figures and Tables

**Table 1:** The experimental design and final results by RSM.

No.	X_1_, Liquid to Solid Ratio (mL/g)	X_2_, Ultrasonic Power (W)	X_3_, Duration of Extraction (min)	Y, AGPL Yield (%)
1	20	600	50	13.36
2	30	600	40	14.94
3	30	600	40	14.86
4	30	600	40	14.75
5	20	600	30	12.93
6	30	750	30	14.42
7	40	600	30	14.61
8	20	450	40	13.71
9	40	750	40	14.26
10	30	450	50	14.07
11	30	450	30	12.86
12	40	450	40	14.72
13	30	600	40	14.95
14	40	600	50	14.79
15	20	750	40	13.50
16	30	750	50	13.66
17	30	600	40	14.90

**Table 2:** ANOVA result of quadratic polynomial regression.

Source	Sum of Squares	D_f_	Mean Square	F-Value	*p*-Value	
Model	7.66	9	0.8512	8.92	0.0043	**
X_1_-Liquid solid ratio	2.98	1	2.98	31.20	0.0018	**
X_2_-Ultrasonic power	0.4095	1	0.4095	23.66	0.0008	**
X_3_-Extraction time	0.1405	1	0.1405	1.47	0.2644	
X_1_X_2_	0.2256	1	0.2256	13.03	0.0086	**
X_1_X_3_	0.0156	1	0.0156	0.1638	0.0153	*
X_2_X_3_	0.9702	1	0.9702	10.17	0.6978	
X_1_^2^	0.4620	1	0.4620	4.84	0.0637	
X_2_^2^	1.06	1	1.06	11.09	0.0126	*
X_3_^2^	1.65	1	1.65	17.31	0.0042	**
Residual	0.6679	7	0.0954			
Lack of Fit	0.0950	3	0.0317	4.83	0.082	
Pure Error	0.0262	4	0.0065			
Cor Total	8.33	16				

Note: * represents significant difference (*p* < 0.05); ** means extremely significant difference (*p* < 0.01); D_f_ stands for degrees of freedom.

**Table 3:** Summary of all statistical parameters of AGPL yield predicted by RSM and GA-ANN.

Model	Process Parameter			AGPL Yield(%)		Coefficient			
	X_1_	X_2_	X_3_	Actual	Predicted	R^2^	MSE	RMSE	AAD (%)
RSM	39.170	595.054	40.736	14.83	14.9049	0.9145	0.0393	0.1982	0.1641
ANN	35.492	696.796	46.124	15.32	15.2822	0.9809	0.0279	0.1669	0.1336

**Table 4:** List of tentatively identified phenolic compounds in the extracts from leaves of *A. grossedentata*.

Compound No.	RT (min)	Formula	Molecular Weight (*m*/*z*)	MS^2^ Fragment Ions (*m*/*z*)	Percentage (%)	Name
**Flavonols**						
1	0.652	C_15_H_14_O_8_	305.0542	259.06; 231.07; 153.02; 149.02; 123.04	1.71%	Leucodelphinidin
2	0.784	C_15_H_14_O_7_	306.0674	259.06; 231.07; 153.02; 123.04	6.74%	Leucocyanidin
3	0.913	C_15_H_12_O_6_	289.0706	243.07; 215.07; 153.02; 107.05	4.29%	Aromadendrin
5	1.683	C_15_H_10_O_7_	303.0491	107.182; 121.173; 151.072; 179.058	2.02%	Quercetin
6	3.116	C_15_H_10_O_6_	287.0523	269.23; 257.23; 187.15	7.80%	Fisetin
7	4.189	C_15_H_12_O_7_	305.1982	259.06; 231.07; 153.02; 149.02	3.10%	Taxifolin
8	4.232	C_15_H_14_O_7_	307.0832	197.25; 169.11; 139.04	10.98%	(−)-Epigallocatechin
9	4.623	C_15_H_14_O_6_	291.0844	197.25; 167.12; 139.04	7.99%	Epicatechin
12	5.176	C_16_H_12_O_6_	299.058	207.48; 165.10; 134.09	2.51%	Kaempferide
19	10.075	C_15_H_12_O_8_	319.04	193.01; 163.14; 125.02	10.37%	Dihydromyricetin
20	10.114	C_21_H_20_O_12_	463.0909	317.03; 316.02	9.22%	Myricitrin
**Flavone glycosides**						
4	1.251	C_17_H_22_O_10_	369.1184	151.06; 133.05; 110.04; 101.03	1.96%	4-O-beta-D-Glucosyl-sinapate
10	4.722	C_27_H_30_O_16_	609.1377	528.21; 301.04; 179.00	6.11%	Quercetin 3-O-rhamnoside 7-O-glucoside
16	7.930	C_21_H_20_O_10_	431.0996	399.22299.21; 153.00; 99.01	2.87%	Apigenin 7-O-beta-D-glucoside
17	8.418	C_21_H_20_O_12_	463.0815	300.03; 271.02; 255.03	2.97%	Quercetin 3-O-glucoside
**Acids**						
11	4.920	C_18_H_30_O_3_	294.4297	277.22; 197.11; 151.11; 107.09	5.38%	Colneleic acid
13	6.088	C_20_H_32_O_2_	303.2413	258.23; 198.14; 59.01	1.92%	Arachidonic acid
14	6.674	C_19_H_38_O_2_	297.2509	279.06; 249.24; 189.03	5.31%	Nonadecanoic acid
15	7.231	C_20_H_28_O_2_	300.2644	282.12; 251.03; 170.10	2.28%	all-trans-Retinoic acid
18	8.901	C_16_H_18_O_9_	353.0882	191.06; 179.04; 173.05; 113.21	5.33%	Neochlorogenic acid

**Table 5:** List of preparation of DESs employed in this study.

NO.	HBA	HBD	Molar Ratio	Water Content	Abbreviation
DES-1	choline chloride	1,2-propylene glycol	1:2	10%	Chcl-Pro
DES-2	choline chloride	malonic acid	1:2	10%	Chcl-Ma
DES-3	choline chloride	urea	1:2	10%	Chcl-Ur
DES-4	choline chloride	1,4-butanediol	1:2	10%	Chcl-Bu4
DES-5	choline chloride	1,3-butanediol	1:2	10%	Chcl-Bu3
DES-6	choline chloride	ethylene glycol	1:2	10%	Chcl-Eg
DES-7	L-carnitine	1,3-butanediol	1:2	10%	Carn-Bu3
DES-8	L-carnitine	urea	1:2	10%	Carn-Ur
DES-9	L-carnitine	1,4-butanediol	1:2	10%	Carn-Bu4
DES-10	L-carnitine	1,2-propylene glycol	1:2	10%	Carn-Pro
DES-11	L-carnitine	ethylene glycol	1:2	10%	Carn-Eg
DES-12	L-carnitine	malonic acid	1:2	10%	Carn-Ma

**Table 6:** Factors and levels of Box–Behnken response surface method.

Variable	Units	Coded Levels			
		Symbol	−1	0	1
Liquid–solid ratio	mL/g	X_1_	20	30	40
Ultrasonic power	W	X_2_	450	600	750
Extraction time	min	X_3_	30	40	50

## Data Availability

Data are contained within the article.
